# Growth-Environment Dependent Modulation of *Staphylococcus aureus* Branched-Chain to Straight-Chain Fatty Acid Ratio and Incorporation of Unsaturated Fatty Acids

**DOI:** 10.1371/journal.pone.0165300

**Published:** 2016-10-27

**Authors:** Suranjana Sen, Sirisha Sirobhushanam, Seth R. Johnson, Yang Song, Ryan Tefft, Craig Gatto, Brian J. Wilkinson

**Affiliations:** School of Biological Sciences, Illinois State University, Normal, Illinois, United States of America; University of Iowa Carver College of Medicine, UNITED STATES

## Abstract

The fatty acid composition of membrane glycerolipids is a major determinant of *Staphylococcus aureus* membrane biophysical properties that impacts key factors in cell physiology including susceptibility to membrane active antimicrobials, pathogenesis, and response to environmental stress. The fatty acids of *S*. *aureus* are considered to be a mixture of branched-chain fatty acids (BCFAs), which increase membrane fluidity, and straight-chain fatty acids (SCFAs) that decrease it. The balance of BCFAs and SCFAs in USA300 strain JE2 and strain SH1000 was affected considerably by differences in the conventional laboratory medium in which the strains were grown with media such as Mueller-Hinton broth and Luria broth resulting in high BCFAs and low SCFAs, whereas growth in Tryptic Soy Broth and Brain-Heart Infusion broth led to reduction in BCFAs and an increase in SCFAs. Straight-chain unsaturated fatty acids (SCUFAs) were not detected. However, when *S*. *aureus* was grown *ex vivo* in serum, the fatty acid composition was radically different with SCUFAs, which increase membrane fluidity, making up a substantial proportion of the total (<25%) with SCFAs (>37%) and BCFAs (>36%) making up the rest. Staphyloxanthin, an additional major membrane lipid component unique to *S*. *aureus*, tended to be greater in content in cells with high BCFAs or SCUFAs. Cells with high staphyloxanthin content had a lower membrane fluidity that was attributed to increased production of staphyloxanthin. *S*. *aureus* saves energy and carbon by utilizing host fatty acids for part of its total fatty acids when growing in serum, which may impact biophysical properties and pathogenesis given the role of SCUFAs in virulence. The nutritional environment in which *S*. *aureus* is grown *in vitro* or *in vivo* in an infection is likely to be a major determinant of membrane fatty acid composition.

## Introduction

*Staphylococcus aureus* is a worldwide significant pathogen in the hospital and the community. Antibiotic resistance has developed in waves [1] such that we now have methicillin-resistant *S*. *aureus* (MRSA), vancomycin-resistant *S*. *aureus* (VRSA) and vancomycin-intermediate *S*. *aureus* (VISA) [2,3]. Given the threat of multiply antibiotic-resistant *S*. *aureus*, various aspects of staphylococcal biology including pathogenicity, antibiotic resistance, and physiology are currently being investigated intensively, in part to support the search for novel anti-staphylococcal agents.

The bacterial cytoplasmic membrane forms an essential barrier to the cell and is composed of a glycerolipid bilayer with associated protein molecules, and is a critical determinant of cell physiology. The biophysical properties of the membrane are to a large extent determined by the fatty acyl residues of membrane phospholipids and glycolipids [4,5]. The lipid acyl chains influence membrane viscosity/fluidity, and impact the ability of bacteria to adapt to changing environments, the passive permeability of hydrophobic molecules, active transport, and the function of membrane-associated proteins [4–6]. Additionally, membrane fatty acid composition has a major influence on bacterial pathogenesis, critical virulence factor expression [7], and broader aspects of bacterial physiology [8].

**Fig 1:**
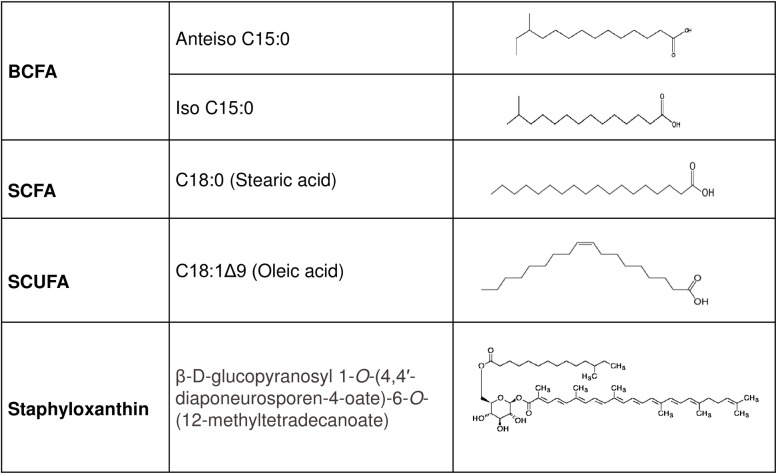
Structures of major fatty acids and staphyloxanthin of the *S*. *aureus* cell membrane.

*S*. *aureus* membrane fatty acids are generally considered to be a mixture of branched-chain fatty acids (BCFAs) and straight-chain fatty acids (SCFAs) [9–11], and for a comprehensive review of earlier literature see [12]. In *S*. *aureus* the major BCFAs are odd-numbered iso and anteiso fatty acids with one methyl group at the penultimate and antepenultimate positions of the fatty acid chains, respectively (Fig 1). BCFAs have lower melting points than equivalent SCFAs and cause model phospholipids to have lower phase transition temperatures [13], and disrupt the close packing of fatty acyl chains [14,15].

**Fig 2:**
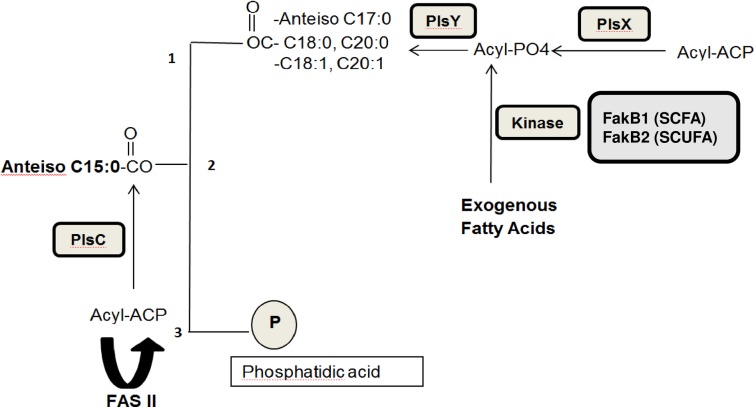
Pathway of phospholipid biosynthesis and the incorporation of exogenous and endogenous fatty acids in *S*. *aureus*.

Fatty acids are major components of the *S*. *aureus* phospholipids, which are phosphatidyl glycerol, cardiolipin and lysysl-phosphatidyl glycerol [16]. BCFAs are biosynthesized from the branched-chain amino acids, isoleucine (anteiso odd-numbered fatty acids), leucine (iso odd-numbered fatty acids), and valine (iso even-numbered fatty acids) via branched-chain aminotransferase and branched-chain α- keto acid dehydrogenase [13]. The branched-chain acyl CoA precursors thus formed are used for the biosynthesis of fatty acids by the dissociated bacterial fatty acid synthesis system (FASII) [5,17]. Phosphatidic acid is a key intermediate in the biosynthesis of the *S*. *aureus* phospholipids [5]. Our current knowledge of the pathway of phospholipid biosynthesis and the incorporation of exogenous and endogenous fatty acids is summarized in Fig 2 [18]. Phosphatidic acid, the universal precursor of phospholipids, is synthesized by the stepwise acylation of *sn*-glycerol-3-phosphate first by PlsY that transfers a fatty acid to the 1-position from acyl phosphate. The 2-position is then acylated by PlsC utilizing acyl-ACP. Acyl-ACP is produced by the FASII pathway and PlsX catalyzes the interconversion of acyl-ACP and acyl phosphate. When *S*. *aureus* is grown in medium that results in a high proportion of BCFAs the major phospholipid, phosphatidyl glycerol, has, almost exclusively, anteiso C17:0 at position 1 and anteiso C15:0 at position 2 [17].

Phosphatidic acid (PtdOH), the universal precursor of phospholipids, is synthesized by the stepwise acylation of *sn*-glycerol-3-phosphate first by PlsY that transfers a fatty acid to the 1-position from acyl phosphate. The 2-position is then acylated by PlsC utilizing acyl-ACP. Acyl-ACP is produced by the FASII pathway and PlsX catalyses the interconversion of acyl-ACP and acyl phosphate. Exogenous fatty acids readily penetrate the membrane and are activated by a fatty acid kinase (FakB1 for SCFAs and FakB2 for SCUFAs) to produce acyl phosphate that can be utilized by PlsY, or that can be converted to acyl-ACP for incorporation into the 2-position by PlsC. Exogenous fatty acids can also be elongated by the FASII pathway. Figure modified from Parsons *et al*. [18].

The membrane lipid composition of *S*. *aureus* is further complicated by the presence of staphyloxanthin, a triterpenoid carotenoid with a C30 chain with the chemical name of α-D-glucopyranosyl-1-*O*-(4,4’-diaponeurosporen-4-oate)-6-*O* (12-methyltetradecanoate) [19](Fig 1). Staphyloxanthin, as a polar carotenoid, is expected to have a significant influence on membrane properties with the expectation that it rigidifies the membrane [20], and Bramkamp and Lopez [21] have suggested that staphyloxanthin is a critical component of lipid rafts in *S*. *aureus* incorporating the organizing protein flotillin. Staphyloxanthin has drawn considerable attention in recent years as a possible virulence factor by detoxifying reactive oxygen species produced by phagocytic cells [22,23], and as a potential target for antistaphylococcal chemotherapy [24].

In our laboratory, we are interested in the mechanisms of action of and resistance to novel and existing anti-staphylococcal antimicrobials [25–27]. Because much antibiotic work employs Mueller-Hinton (MH) medium, [28] we had occasion to determine the fatty acid composition of a *S*. *aureus* strain grown in this medium. The analysis was carried out using the MIDI microbial identification system (Sherlock 4.5 microbial identification system; Microbial ID, Newark, DE, USA), [29]. We were taken aback when the fatty acid profile came back showing a very high percentage (84.1%) of BCFAs, and the organism was not even identified by MIDI as a *S*. *aureus* strain. In a previous study where we grew *S*. *aureus* in BHI broth we found that 63.5% of the fatty acids were BCFAs, and 32.4% were SCFAs [10]. This is a much more typically observed balance between BCFAs and SCFAs in previous studies of the fatty acid composition of *S*. *aureus* [9–12].

A range of different media are used for cultivating *S*. *aureus* in studies from different laboratories [30]. These are mostly complex media such as Tryptic Soy Broth (TSB), BHI broth, MH broth, Luria-Bertani (LB) broth, and, much more rarely, defined media [11]. Ray *et al*. [30] and Oogai *et al* [31] have pointed out that different media have major, but largely unstudied and ignored, effects on the expression of selected target virulence and regulatory genes. Although seemingly prosaic at first glance, issues of choice of strain and medium are nevertheless critical considerations in staphylococcal research [32]. These authors [32], in their recent protocol publication on the growth and laboratory maintenance of *S*. *aureus*, have suggested that TSB and BHI media are the media of choice for staphylococcal research. In light of recent literature in various microorganisms, it is becoming evident that environment has a tremendous effect on the physiology of different pathogens; hence cells from *in vivo* growth are significantly different from *in vitro* cultured ones. Such distinctions are likely important for studying antimicrobial susceptibilities, drug resistances and pathogenesis.

We decided to carry out a systematic study of the impact of growth medium on the fatty acid and carotenoid composition of *S*. *aureus* given the large potential impact of these parameters on membrane biophysical properties and its further ramifications. The BCFA: SCFA ratio was significantly impacted by the laboratory medium used, with media such as MH broth encouraging high proportions of BCFAs. However, strikingly, when cells were grown in serum, an *ex vivo* environment, the fatty acid composition changed radically, with straight-chain unsaturated fatty acids (SCUFAs) (Fig 1), which were not detected in cells grown in laboratory media, making up a major proportion of the total fatty acids. This extreme plasticity of *S*. *aureus* membrane lipid composition is undoubtedly important in determining membrane physical structure and thereby the functional properties of the membrane. The alterations in the fatty acid composition as a result of interactions of the pathogen with the host environment may be a crucial factor in determining its fate in the host. Typically used laboratory media do not result in a *S*. *aureus* membrane fatty acid composition that closely resembles the likely one of the organism growing *in vivo* in a host.

## Materials and Methods

### Bacterial Strains and Growth Conditions

The primary *S*. *aureus* strains studied were strain JE2 derived from strain LAC USA300 [33] and strain SH1000. USA300 strain JE2 is a prominent community-acquired MRSA lineage, which is a leading cause of aggressive cutaneous and systemic infections in the USA [1,34,35]. Strain JE2 has a well-constructed diverse transposon mutant library [33]. *S*. *aureus* strain SH1000, is an 8325-line strain that has been used extensively for many years in genetic and pathogenesis studies [36]. The laboratory media used were MH broth, TSB, BHI broth and LB from Difco. For growth and fatty acid composition studies cultures of *S*. *aureus* strains were grown at 37°C in 250 ml Erlenmeyer flasks containing each of the different laboratory media with a flask–to-medium volume ratio of 5:1. Growth was monitored by measuring the OD_600_ at intervals using a Beckman DU-65 spectrophotometer.

### Growth of *S*. *aureus* in Serum

Sterile fetal bovine serum of research grade was purchased from Atlanta Biologics, USA. The aliquoted serum was incubated in a water bath at 56°C for 30 min to heat inactivate the complement system. *S*. *aureus* cells were grown for 24 hours in 50 ml of serum in a 250 ml flask at 37°C with shaking at 200 rpm.

### Analysis of the Membrane Fatty Acid Composition of *S*. *aureus* Grown in Different Media

The cells grown in the different conventional laboratory media were harvested in mid-exponential phase (OD_600_ 0.6), and after 24 hrs of growth in serum, by centrifugation at 3000 x g at 4°C for 15 minutes and the pellets were washed three times in cold distilled water. The samples were then sent for fatty acid methyl ester (FAME) analysis whereby the fatty acids in the bacterial cells (30–40 mg wet weight) were saponified, methylated, and extracted. The resulting methyl ester mixtures were then separated using an Agilent 5890 dual-tower gas chromatograph and the fatty acyl chains were analyzed and identified by the MIDI microbial identification system (Sherlock 4.5 microbial identification system) at Microbial ID, Inc. (Newark, DE) [29]. The percentages of the different fatty acids reported in the tables and figures are the means of the values from three separate batches of cells under each condition. Some minor fatty acids such as odd-numbered SCFAs were not reported.

### Extraction and Estimation of Carotenoids

For quantification of the carotenoid pigment in the *S*. *aureus* cells grown in different media, the warm methanol extraction protocol was followed as described by Davis *et al*. [37]. Cultures of *S*. *aureus* were harvested at mid-exponential phase and were washed with cold water. The pellets were then extracted with warm (55°C) methanol for 5 min. The OD_465_ of the supernatant after centrifugation was measured using a Beckman DU 70 spectrophotometer. Determinations were carried out in triplicate. Significant differences between carotenoid content of *S*. *aureus* grown in different media were determined by analysis of variance (ANOVA) using SAS 9.4 (SAS Institute, NC) with post hoc Tukey’s test.

### Measurement of the Fluidity of the *S*. *aureus* Membrane

The fluidities of the cell membrane of the *S*. *aureus* strains grown in different media were determined by anisotropic measurements using the fluorophore diphenylhexatriene (DPH) following the protocol described previously [38]. Mid exponential phase cells grown in respective media and serum were harvested and washed with cold sterile PBS (pH 7.5). The pellets were then resuspended in PBS containing 2 μM DPH (Sigma, MO) to an OD_600_ of about 0.3 and incubated at room temperature in the dark for 30 min. Fluorescence polarization emitted by the fluorophore was measured using a PTI Model QM-4 Scanning Spectrofluorometer at an excitation wavelength of 360 nm and emission wavelength of 426 nm. The experiments were performed with three separate fresh batches of cells. Significant differences between mean polarization values of *S*. *aureus* grown in different media were determined by analysis of variance (ANOVA) using SAS 9.4 (SAS Institute, NC) with post hoc Tukey’s test.

## Results

The two main strains studied were USA300 strain JE2 and strain SH1000. The genome sequences of both strains are known. The USA300 JE2 background represents the most prominent community-associated methicillin resistance lineage in the US, and is the strain in which the Nebraska Transposon Mutant Library is constructed [33]. Strain SH1000 is an 8325-line strain in which the defect in SigB has been corrected [39], and for many years 8325-line strains have been used as model strains in genetic studies of staphylococcal pathogenesis [36]. These strains were chosen for their significance as pathogens, well-developed knowledge of their genetics, physiology and virulence, and familiarity to the staphylococcal research community.

### MH broth and LB Increase the Content of BCFAs and TSB and BHI Broth Increase the Content of SCFAs

**Fig 3:**
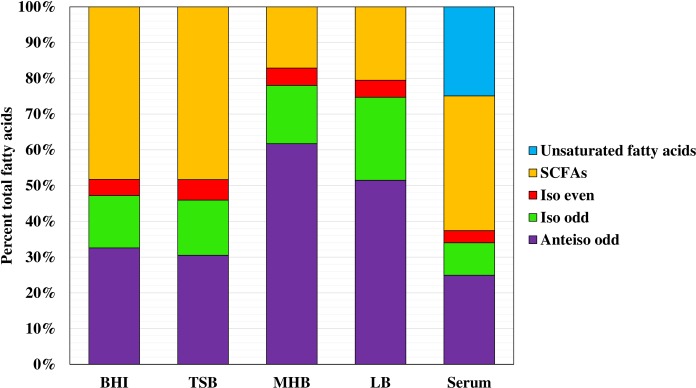
Membrane fatty acid composition of *S*. *aureus* strain JE2 cells grown in different media. Membrane fatty acid composition of log phase *S*. *aureus* strain JE2 cells grown in BHI, TSB, MH broth, LB and fetal bovine serum were summarized into the various common classes of fatty acids. Figure shows representative data from at least three independent experiments.

The fatty acid compositions of strain JE2 grown in different laboratory media are shown in Fig 3 and in more detail in S1 Table. Growth in MH broth and LB resulted in a high content of BCFAs, 80.9% and 77.2% respectively, whereas SCFAs were 19.1% and 22.8% respectively. However, in TSB and BHI broth the BCFAs contents were lower at 51.7% and 51.5% respectively, and SCFAs were increased to 48.3 and 48.5% respectively. In MH broth anteiso odd-numbered fatty acids were the major fatty acids in the profile (59.8%), followed by even-numbered SCFAs (16.6%), iso odd-numbered fatty acids (15.8%), with iso even-numbered fatty acids making up only a minor portion (4.7%). Anteiso C15:0 was the predominant fatty acid in the membrane lipids (39%). This particular fatty acid has a significant impact on fluidizing membranes [40,41]. The anteiso fatty acids were significantly reduced in TSB-grown cells (29.3%). The major SCFAs in TSB-grown cells were C18:0 and C20:0 at 19.1% and 18.6% respectively. Overall, the fatty acid compositions were in line with many previous studies of *S*. *aureus* fatty acid composition [9–12], but we are unaware of previous studies that have identified this impact of medium on the proportions of BCFAs and SCFAs in the membrane.

**Fig 4:**
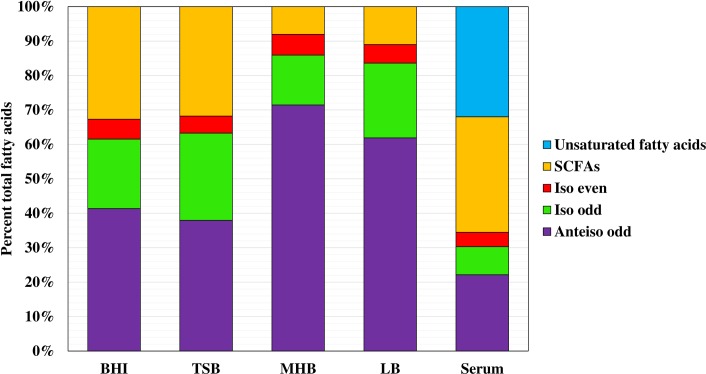
Membrane fatty acid composition of *S*. *aureus* strain SH1000 cells grown in different media. Membrane fatty acid composition of log phase *S*. *aureus* strain SH1000 cells grown in BHI, TSB, MH broth, LB and fetal bovine serum were summarized into the various common classes of fatty acids. Figure shows representative data from at least three independent experiments.

The results of a similar series of experiments with strain SH1000 are shown in Fig 4 and S2 Table. In strain SH1000 the BCFAs were higher than JE2 in all media- BHI 66.6%, TSB 68.5%, with particularly high contents in MH broth, 90.2%, and LB, 89%. The proportion of SCFAs was correspondingly smaller in all cases compared to strain JE2. Anteiso fatty acids were the major class of fatty acids in all media, amongst which anteiso C15:0 was present in the highest amount in all cases. However, the same phenomenon was noted where MH broth and LB encouraged a high proportion of BCFAs, low SCFAs, and TSB and BHI had the opposite effects on fatty acid composition. Two additional media were studied with this strain. Both Tryptone Broth [42] and defined medium [43] resulted in high BCFAs (80.4% and 85% respectively), and low SCFAs (19.7% and 15% respectively).

### The Fatty Acid Composition of *S*. *aureus* Grown *ex vivo* in Serum is Radically Different to Those of the Organism Grown in Laboratory Media

It was of interest to try and get an idea of the fatty acid composition of *S*. *aureus* grown *in vivo*. Strain JE2 and SH1000 were grown *ex vivo* in serum, which resulted in major changes in the fatty acid profile (Figs 3 & 4 and S1 and S2 Tables). Total BCFAs were reduced to 37.5% in JE2 and 36.3% in SH1000; SCFAs were at 37.8% in JE2 and 32.1% in SH1000, but 25% of the fatty acid profile in the case of JE2 and 30.6% in SH1000 was accounted for by SCUFAs. Strikingly, this type of fatty acid was not present in the profile of the organism when grown in laboratory media. Interestingly, BCFAs and SCUFAs have similar effects in increasing fluidity of the membrane [4].

### Carotenoid Content of Cells Grown in Different Media

**Fig 5:**
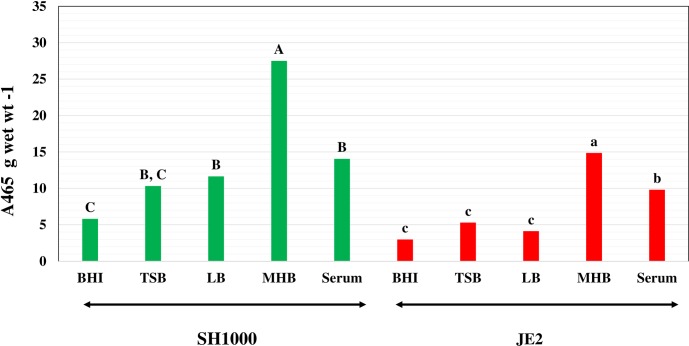
Influence of growth environment on the carotenoid content of *S*. *aureus*. The strains, JE2 (red columns) and SH1000 (green columns), were grown in different growth media and the carotenoid content was estimated after extraction by warm methanol. Different letters indicate significant differences in the carotenoid content.

Staphyloxanthin is another significant membrane component that might impact the biophysical properties of the membrane. Accordingly, the carotenoid contents of cells grown in different media were determined and the results are shown in Fig 5. Strain SH1000 cells grown in MH broth had a much higher carotenoid content than cells grown in the other media. The pellets of cells grown in this particular media were noticeably yellow. It is possible that the carotenoid content rises to counterbalance the potentially high fluidity of MH broth-grown cells with their high content of BCFAs, specifically mainly anteiso fatty acids. MH broth (high BCFAs) and serum (high SCUFAs)—grown cells had higher carotenoid contents than TSB or BHI broth–grown cells as revealed by statistical analysis of the data (Fig 5), which demonstrated that the carotenoid content of cells grown in MH broth was distinctly different from cells grown in other media. In strain JE2 MH broth- and serum-grown cells also had higher carotenoid contents than did cells grown in BHI broth, TSB or LB. In general, this strain was less pigmented than strain SH1000. Statistical analysis of *S*. *aureus* strain JE2 carotenoid content showed that cells grown in MH broth and serum were placed in the same group, and the cells grown in BHI broth, LB and TSB were in a different group.

### Membrane Fluidity of *S*. *aureus* Cells Grown in Different Media

**Fig 6:**
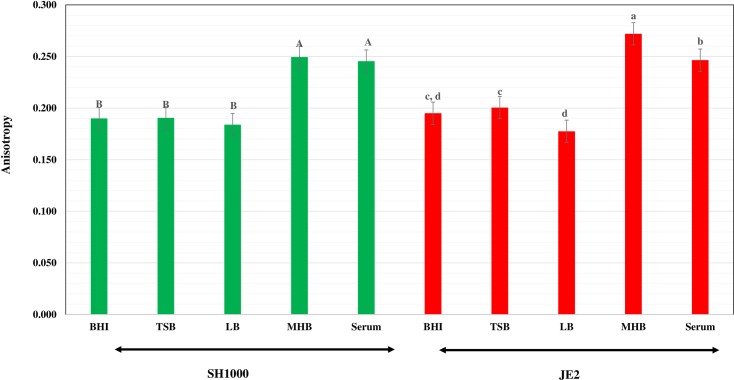
Influence of growth environment on the membrane fluidity of *S*. *aureus* cells. The strains, JE2 (red columns) and SH1000 (green columns), were grown in the different media to mid exponential phase and membrane anisotropy was measured by fluorescence polarization. Different letters indicate statistically significant differences in the fluorescence polarization values.

The membrane fluidity of cells of strain SH1000 grown in BHI broth, LB and TSB were very similar (0.185–0.19) as shown in Fig 6. The membranes of MH-broth and serum-grown cells, 0.25 and 0.248 were significantly less fluid than cells grown in the other media. Possibly the higher carotenoid contents of cells grown in MH broth and serum rigidify the membrane. Strain JE2 also showed a similar pattern of membrane fluidity in the different growth media (Fig 6). The membrane fluidity of both strains was highest in cells grown in LB, consistent with the high content of BCFAs. Furthermore, in this medium there was no accompanying increase in staphyloxanthin content with its possible membrane rigidifying effect in contrast to what was observed in MH broth or serum-grown cells. Statistical analysis showed that the fluorescence polarization values of both *S*. *aureus* strain JE2 and SH1000 grown in MH broth and serum were significantly different from the cells grown in BHI broth, TSB and LB as indicated in Fig 5.

## Discussion

From numerous studies over the past several decades of *S*. *aureus* grown *in vitro* in various laboratory media it is considered that the membrane fatty acid composition of the organism is a mixture of BCFAs and SCFAs [9–12], and BCFAs have generally been found to be predominant. Through study of a range of different conventional growth media, we found that certain media encouraged a higher proportion of BCFAs than others, whereas in some media the proportion of SCFAs was increased. This may have significant physiological ramifications given the opposing effects of BCFAs and SCFAs on membrane fluidity with BCFAs fluidizing and SCFAs rigidifying the membrane [4]. However, there was a radical change in the entire fatty acid composition when the organism was grown *ex vivo* in serum with SCUFAs appearing in the profile in significant amounts accompanied by a decrease in BCFA content.

### What Determines the Balance Between BCFAs and SCFAs in Cells Grown in Laboratory Media?

MH medium leads to a high proportion of BCFAs in the staphylococcal cells, whereas growth in TSB leads to an increase in the proportion of SCFAs. MH broth (Difco) is composed of beef extract powder (2 g/l), acid digest of caseine (17.5 g/l), and soluble starch (1.5 g/l). Thus, by far the major medium component is acid digest of caseine, and this is expected to be high in free amino acids. TSB (Difco) is composed of pancreatic digest of caseine (17 g/l), enzymatic digest of soybean meal (3 g/l), dextrose (2.5 g/l), sodium chloride (5 g/l) and dipotassium phosphate (2.5 g/l). The major components then of TSB are a mixture of peptides formed by enzymatic digestion of caseine and soybean meal. Payne and Gilvarg [44] fractionated Bacto Neopeptone using gel filtration. They found that peptides with a molecular weight below 650 represented about 25% of the mixture, and free amino acids were about 1% of the entire preparation. We believe that the free amino acids from the acid digest of caseine can have a dominant effect on the fatty acid composition. Inclusion of isoleucine, leucine, or valine in the growth medium of *Listeria monocytogenes* results in large increases in fatty acids derived from the particular amino acid in question [29,45]. The BCFA content of *S*. *aureus* is lower in *S*. *aureus* grown in TSB, where pool amino acids are likely to be mainly derived from transported peptides [46], than when grown in defined medium, which probably gives rise to higher pool levels of amino acids [47] and this work. Mutants of *S*. *aureus* in the transporters of leucine and valine lacked odd- and even-numbered fatty acids derived from these amino acids when grown in defined medium [47].

Growth in media such as TSB and BHI broth lead to higher proportions of SCFAs than media such as MH broth, although SCUFAs were not detected. The origin of SCFAs is not clear as to whether they originate from the medium or are biosynthesized. Typically, in bacteria SCFAs are biosynthesized from acetyl CoA via the activities of FabH and the FASII system. However, acetyl CoA was a poor substrate for *S*. *aureus* FabH [48], whereas the enzyme had high activity for butyryl CoA, raising the possibility that butyrate is the primer for biosynthesis of SCFAs in *S*. *aureus*. It is also possible that SCFAs that may be present in TSB and BHI may be utilized directly for fatty acid elongation to the SCFAs in the membrane typical of growth in these media.

### The Underappreciated Ability of *S*. *aureus* to Incorporate Host Fatty Acids From Serum

A striking finding in our paper is that *S*. *aureus* has the capacity to incorporate large proportions of SCFAs and SCUFAs when grown *ex vivo* in serum. Earlier reports of fatty acid composition have not reported significant amounts of SCUFAs in *S*. *aureus* [9–12]. Indeed, it appears that *S*. *aureus* lacks the genes necessary to biosynthesize unsaturated fatty acids [18]. An early report by Altenbern [49] showed that inhibition of growth by the fatty acid biosynthesis inhibitor cerulenin could be relieved by SCFAs or SCUFAs, implying *S*. *aureus* had the ability to incorporate preformed fatty acids. Fatty acid compositional studies of the cells were not reported though. Serum is lipid rich [50–52] and a comprehensive analysis of the human serum metabolome including lipids has recently been published [53]. BCFAs are present, if at all, in only very small amounts in serum. Bacterial pathogens typically have the ability to incorporate host-derived fatty acids thereby saving carbon and energy since fatty acids account for 95% of the energy requirement of phospholipid biosynthesis [54]. Exogenous fatty acids readily penetrate the membrane and are activated by a fatty acid kinase to produce acyl phosphate that can be utilized by PlsY for incorporation into the 1 position of the glycerol moiety of phospholipids, or they can be converted to acyl-ACP for incorporation into the 2 position by PlsC (Fig 2) [18].

The FASII pathway has been considered to be a promising pathway for inhibition with antimicrobial drugs. The viability of FASII as a target for drug development was challenged by Brinster *et al*. [55] especially for bacteria such as streptococci where all the lipid fatty acids could be replaced by SCFAs and SCUFAs from serum. However, Parsons *et al*. [17] showed that exogenous fatty acids could only replace about 50% of the phospholipid fatty acids in *S*. *aureus* and concluded that FASII remained a viable drug target in this organism.

Besides occurring in membrane phospholipids, fatty acids are present in staphyloxanthin, glycolipids and lipoteichoic acid and in lipoproteins at their N terminus in the form of an N-acyl-S-diacyl-glycerol cysteine residue and an additional acyl group amide linked to the cysteine amino group [56]. It is estimated that there are 50–70 lipoproteins in *S*. *aureus*, and many of them are involved in nutrient acquisition. The distribution of growth environment-derived SCFAs and SCUFAs in these lipid molecules has not yet been examined.

### Changes in Staphyloxanthin in Cells Grown Under Different Conditions with Different Membrane Fatty Acid Compositions

The carotenoid staphyloxanthin is a unique *S*. *aureus* membrane component that affects membrane permeability, defense against reactive oxygen species, and is a potential drug target. It appeared that cells grown in media encouraging a high proportion of BCFAs or in serum resulting in high SCUFAs, both of which would be expected to increase membrane fluidity, tended to have higher staphyloxanthin contents. Cells grown in MH broth or serum had cellular membranes that were less fluid that may be attributable to the higher content of staphyloxanthin. However, this relationship is likely to be complex in that LB-grown cells that had high BCFAs did not have high carotenoid levels, and the phenomenon is deserving of more detailed investigation. Interestingly, in the biosynthesis of staphyloxanthin, the end step involves an esterification of the glucose moiety with the carboxyl group of anteiso C15:0 by the activity of the enzyme acyltransferase CrtO [19]. It is not known whether anteiso C15:0 can be replaced by SCUFAs.

### Plasticity of *S*. *aureus* Membrane Lipid Composition and its Possible Ramifications in Membrane Biophysics and Virulence

Given the crucial role of the biophysics of the membrane in all aspects of cell physiology, such radical changes in the membrane lipid profile can have significant but as yet undocumented impacts on critical functional properties of cells such as virulence factor production, susceptibilities to antimicrobials and tolerance of host defenses. It is important to assess the biophysical and functional properties of the membranes of the cells with such radically different fatty acid compositions.

The susceptibilities of the strains to three antibiotics designated as hydrophobic, oxacillin, vancomycin and rifampicin and three designated as hydrophilic, chloramphenicol, penicillin G, and tetracycline [57], were determined on TSB, BHI, MH, LB and serum agar plates by disk diffusion. There was no striking difference in antibiotic susceptibilities between the different artificial media. Zones of inhibition were markedly lower in both strains on serum agar for rifampicin, chloramphenicol, and tetracycline. However, membrane permeability studies clearly need to be done in a more simplified system such as lipid vesicles of defined fatty acid composition [58] to simplify the interpretation of any differences observed. Cells grown in serum had higher hemolytic activity, and MH broth and serum- grown cells had lower autolytic activities than cells grown in the other media (unpublished observations).

Although BCFAs and SCUFAs both increase membrane fluidity, they do not yield cells with identical morphologies [15], or fitness for tolerating cold stress [59]. Also a *S*. *aureus* fatty acid auxotroph created by inactivation of acetyl coenzyme A carboxylase (*ΔaccD*) was not able to proliferate in mice, where it would have access to SCFAs and SCUFAs [60]. Due to the ability of a pathogen to adapt and undergo dramatic alterations when subjected to a host environment, there is a growing appreciation in the research community for the fact that the properties of the organism grown *in vivo* are probably very different from when it is grown *in vitro*. This distinction may have a huge impact on critical cellular attributes controlling pathogenesis and resistance to antibiotics. Expression of virulence factors is significantly different in serum-grown organisms [31], and there are global changes in gene expression when *S*. *aureus* is grown in blood [61]. *S*. *aureus* grown in serum or blood will have different membrane lipid compositions than cells grown in laboratory media and this may have a significant impact on the expression of virulence factors and pathogenesis of the organism.

The relationship between *S*. *aureus* and long-chain SCUFAs and SCFAs is a complex one. On one hand these fatty acids in the skin and other tissues form part of the innate defense system of the host due to their antimicrobial activities [62–64]. Very closely related structures can either be inhibitory to growth at low concentrations, or can have little effect on growth at relatively high concentrations [42,65–67]. For example, C16:1Δ6 and C16:1Δ9 are highly inhibitory whereas C18:1Δ9 and C18:0 are not inhibitory and are actually incorporated into the phospholipids by this pathogen [42].

The enzyme fatty acid kinase (Fak) responsible for incorporation of extracellular fatty acids into *S*. *aureus* phospholipids [18], is also a critical regulator of virulence factor expression [68], and biofilm formation [69]. Fak phosphorylates extracellular fatty acids for incorporation into *S*. *aureus* membrane phospholipids [18] (Fig 2). FakA is a protein with an ATP-binding domain that interacts with FakB1 and FakB2 proteins that bind SCFAs and SCUFAs preferentially respectively. Fak activity producing acyl phosphates was proposed to be involved in the control of virulence gene expression. Interestingly FakB2 shows a high degree of specificity for C18:1Δ9, a fatty acid not produced by *S*. *aureus*, that may act as a sensor for the host environment through its abundance in the host [18], which is subsequently incorporated into the membrane lipids.

Additionally, fatty acids are important components of lipoproteins that contribute important microbe-associated molecular patterns that bind to Toll-like receptors and activate innate host defense mechanisms. Recently, Nguyen *et al*. [70] have shown that when *S*. *aureus* is fed SCUFAs they are incorporated into lipoproteins and the cells have an increased toll-like receptor 2- dependent immune stimulating activity, which enhances recognition by the immune defense system.

## Concluding Remarks

We have demonstrated a hitherto poorly recognized growth environment-dependent plasticity of *S*. *aureus* membrane lipid composition. The balance of BCFAs and SCFAs was affected significantly by the variations in laboratory medium in which the organism grew. SCUFAs became a major membrane fatty acid component when the organism was grown in serum. These findings speak to the properties of pathogens grown *in vitro* versus *in vivo*. In 1960 Garber [71] considered the host as the growth medium and the importance of the properties of the pathogen at the site of infection. There has been a renewed appreciation of this in recent years [72]. Massey *et al*. [73] showed that *S*. *aureus* grown in peritoneal dialysate acquired a protein coat. Krismer *et al*. [74] devised a synthetic nasal secretion medium for growth of *S*. *aureus*. However, Chaves Moreno *et al*. [75] determined the *in vivo* metatranscriptome of *S*. *aureus* by RNAseq analysis of RNA isolated from the anterior nares of documented *S*. *aureus* carriers. *In vitro* transcriptomes did not mimic *in vivo* transcriptomes. Citterio *et al*. [76] reported that the activities of antimicrobial peptides and antibiotics were enhanced against various pathogenic bacteria by supplementation of the media with blood plasma to mimic *in vivo* conditions.

*S*. *aureus* may be the most versatile of all pathogens causing diseases ranging from superficial skin infections to deep seated disseminated diseases of various organs and tissues. The organism forms biofilms on tissues, intravenous catheters and prosthetic devices. *S*. *aureus* can thrive in multiple heterogenous environments. We propose that the nutritional environment is the main determinant of membrane fatty acid composition. If SCUFAs are present in the environment these will be preferentially incorporated into the lipids to a tolerated extent, although there appears to be a requirement for a significant proportion of biosynthesized anteiso odd-numbered fatty acids. It is sobering to realize that the vast majority of studies of staphylococcal biology utilizing organisms grown in artificial media have been carried out with cells lacking SCUFAs in their membrane.

## Supporting Information

S1 Table The membrane fatty acid profile of *S*. *aureus* strain JE2 grown in various conventional media and in serum. (PDF)

S2 Table The membrane fatty acid composition of *S*. *aureus* strain SH1000 grown in various conventional media and in serum (PDF)
