# Supplementary material for: Growth-Environment Dependent Modulation of Staphylococcus aureus Branched-Chain to Straight-Chain Fatty Acid Ratio and Incorporation of Unsaturated Fatty Acids
Source: PLoS One. 2016 Oct 27;11(10):e0165300. doi: 10.1371/journal.pone.0165300 (PMC5082858; doi:10.1371/journal.pone.0165300)
Supplement: S1 Table — (PDF) [file pone.0165300.s001.pdf]

Supporting information

S1 Table. The membrane fatty acid profile of *S. aureus* strain JE2 grown in various conventional media and in serum

% (wt/wt) of total fatty acids

**Membrane fatty acids**		**BHI**	**TSB**	**MHB**	**LB**	**Serum**
Anteiso odd	C15:0	28.5	26.9	39	36.5	21
	C17:0	2.6	2.4	15	11	4
	C19:0	ND	ND	5.8	2.4	ND
Iso odd	C15:0	12.2	12.8	7.7	13.3	7.1
	C17:0	1.8	2	4.9	6.6	2
	C19:0	ND	ND	3.2	2.6	0
Iso even	C14:0	2.9	3.7	1	1.4	1.7
	C16:0	1.4	1.8	2.1	2.2	1.7
	C18:0	ND	ND	1.6	1	ND
Straight even	C14:0	3.3	2.3	1.1	0	1.7
	C16:0	7.9	6.4	1.8	3	16.4
	C18:0	21.5	19.1	6.5	10	13.3
	C20:0	13.4	18.6	7.2	6.9	4.8
Unsaturated fatty acids	C16:1Δ9	ND	ND	ND	ND	1.7
	C18:1Δ9	ND	ND	ND	ND	16
	C18:1Δ7	ND	ND	ND	ND	4.2
	C20:1Δ9	ND	ND	ND	ND	2.1
	C20:4Δ6,9,12,15	ND	ND	ND	ND	1
BCFAs		49.4	49.6	80.3	77	37.5
SCFAs		46.1	46.4	16.6	19.9	37.8
SCUFAs		ND	ND	ND	ND	25

ND- Not detected
