# Supplementary material for: Growth-Environment Dependent Modulation of Staphylococcus aureus Branched-Chain to Straight-Chain Fatty Acid Ratio and Incorporation of Unsaturated Fatty Acids
Source: PLoS One. 2016 Oct 27;11(10):e0165300. doi: 10.1371/journal.pone.0165300 (PMC5082858; doi:10.1371/journal.pone.0165300)
Supplement: S2 Table — (PDF) [file pone.0165300.s002.pdf]

Supporting information

S2 Table. The membrane fatty acid composition of *S. aureus* strain SH1000 grown in various conventional media and in serum

% (wt/wt) of total fatty acid

**Membrane fatty acids**		**BHI**	**TSB**	**MHB**	**LB**	**Serum**
Anteiso odd	C15:0	33.6	31.6	43.7	42	18.2
	C17:0	6.4	6	20.7	16.8	3
	C19:0	1	ND	5.7	3.1	ND
Iso odd	C15:0	15.7	18.7	6.8	12.2	6
	C17:0	4.3	5.2	5.1	7.2	1.8
	C19:0	ND	1.2	2.3	2.3	ND
Iso even	C14:0	2.6	2.2	1.2	1.3	2.4
	C16:0	3.1	2.7	3.1	2.9	1.6
	C19:0	ND	ND	1.6	1.2	ND
Straight even	C14:0	2.7	2	ND	ND	1.1
	C16:0	8.1	7.8	1.4	2.4	13.2
	C18:0	15.2	15.4	4.2	6	12.1
	C20:0	6.4	6.3	2.3	2.6	4.4
Unsaturated fatty acids	C16:1Δ9	ND	ND	ND	ND	1.5
	C18:1Δ9	ND	ND	ND	ND	15.4
	C18:1Δ7	ND	ND	ND	ND	6.4
	C20:1Δ9	ND	ND	ND	ND	5
	C20:4Δ6,9,12,15	ND	ND	ND	ND	2.3
BCFAs		66.7	67.6	90.2	89	33
SCFAs		32.4	31.5	7.9	11	32.1
SCUFAs		ND	ND	ND	ND	30.6

ND- Not detected
